# Age dependence of the rapid antidepressant and synaptic effects of acute NMDA receptor blockade

**DOI:** 10.3389/fnmol.2014.00094

**Published:** 2014-12-01

**Authors:** Elena Nosyreva, Anita E. Autry, Ege T. Kavalali, Lisa M. Monteggia

**Affiliations:** Department of Neuroscience, University of Texas Southwestern Medical CenterDallas, TX, USA

**Keywords:** antidepressant, ketamine, behavior, synaptic potentiation, development

## Abstract

Ketamine is a *N*-methyl-D-aspartate receptor (NMDAR) antagonist that produces rapid antidepressant responses in individuals with major depressive disorder. The antidepressant action of ketamine has been linked to blocking NMDAR activation at rest, which inhibits eukaryotic elongation factor 2 kinase leading to desuppression of protein synthesis and synaptic potentiation in the CA1 region of the hippocampus. Here, we investigated ketamine mediated antidepressant response and the resulting synaptic potentiation in juvenile animals. We found that ketamine did not produce an antidepressant response in juvenile animals in the novelty suppressed feeding or the forced swim test. In addition ketamine application failed to trigger synaptic potentiation in hippocampal slices obtained from juvenile animals, unlike its action in slices from adult animals. The inability of ketamine to trigger an antidepressant response or subsequent synaptic plasticity processes suggests a developmental component to ketamine mediated antidepressant efficacy. We also show that the NMDAR antagonist AP5 triggers synaptic potentiation in mature hippocampus similar to the action of ketamine, demonstrating that global competitive blockade of NMDARs is sufficient to trigger this effect. These findings suggest that global blockade of NMDARs in developmentally mature hippocampal synapses are required for the antidepressant efficacy of ketamine.

## INTRODUCTION

Major depressive disorder (MDD) is a serious mental condition in which there is a need for fast acting treatment. Ketamine, a non-competitive *N*-methyl-D-aspartate receptor (NMDAR) antagonist has been shown to elicit a rapid antidepressant response in patients with MDD (Berman et al., 2000; Zarate et al., 2006; Price et al., 2009) and bipolar depression (Diazgranados et al., 2010a; Zarate et al., 2012). Ketamine has also been reported to have rapid anti-suicidal effects (Price et al., 2009; DiazGranados et al., 2010b; Larkin and Beautrais, 2011; Zarate et al., 2012), an area of critical unmet need. However, ketamine does not present an ideal profile as an antidepressant due to its potential psychotomimetic effects as well as abuse potential. Thus, there has been interest in delineating the cellular mechanisms that underlie ketamine action to facilitate development of safer compounds with similar robust and rapid antidepressant effects.

In recent work, the antidepressant effect of ketamine have been recapitulated in animal models predictive of antidepressant efficacy (Maeng et al., 2008; Li et al., 2010; Autry et al., 2011; Nosyreva et al., 2013; Gideons et al., 2014), such as the forced swim test (FST), the novelty suppressed feeding (NSF) test, and learned helplessness test. In parallel, in hippocampal slices we have also observed a form of synaptic potentiation elicited after 30-min application of ketamine in the absence of stimulation. After ketamine application at rest, we stimulated the Schaffer collateral/commissural afferents and recorded postsynaptic responses from CA1 dendrites. These responses were significantly enhanced after ketamine treatment in adult rats and mice (Autry et al., 2011; Nosyreva et al., 2013). The behavioral as well as the synaptic effects of ketamine are dependent on the function of eukaryotic elongation factor 2 (eEF2) kinase and protein synthesis as ketamine failed to elicit a behavioral response or potentiation on the eEF2 kinase null mice as well as after application of protein translation blocker anisomycin (Autry et al., 2011; Nosyreva et al., 2013). Although these studies have provided a strong correlation between the synaptic action of ketamine and its rapid antidepressant effects, they have not yet addressed whether this effect is dependent on the stage of synapse development. Synaptic plasticity related processes are age dependent and their properties vary during synapse development. For instance, NMDAR only postsynaptically silent synapses are more prevalent during earl stages of synapse development (Wu et al., 1996; Hanse et al., 2013). We therefore examined whether ketamine elicits an antidepressant response and triggers similar synaptic potentiation in juvenile animals to that previously observed in adults. In addition, these earlier studies have not addressed whether the synaptic effect of ketamine and another use-dependent NMDAR blocker MK-801 could be mimicked by the widely used competitive non-use dependent NMDAR antagonist D-AP5 [D-AP5 (2*R*)-amino-5-phosphonopentanoate].

In this study, we report that ketamine does not trigger a behavioral antidepressant response in juvenile animals as it does in adults. We also find that ketamine does not trigger synaptic potentiation in juvenile animals, demonstrating that the antidepressant and synaptic effects of ketamine require the establishment of mature synaptic contacts. In addition, we show that the competitive NMDAR antagonist D-AP5, elicits synaptic potentiation in developmentally mature slices complementing the earlier observation that the competitive NMDAR antagonist 3-[(*R*)-2-Carboxypiperazin-4-yl]-propyl-1-phosphonic acid (CPP) triggers a rapid antidepressant response *in vivo* (Autry et al., 2011).

## MATERIALS AND METHODS

### ANIMALS

Male C57BL/6 mice from Jackson Labs and male Sprague–Dawley rats from Charles River were obtained. For the experimental paradigms, mice were 4 weeks of age and rats were either 2–3 (developmentally immature/juvenile) or 6–8 (developmentally mature, young adult) weeks of age (McCutcheon and Marinelli, 2009). Animals were maintained on a 12-h light/dark cycle with *ad libitum* access to food and water, except when indicated. All experiments were conducted and analyzed blind to treatment group. Experiments were approved by the Institutional Animal Care and Use Committee at the UT Southwestern Medical Center.

### DRUG TREATMENT

Ketamine (Fort Dodge Animal Health) and D-AP5 [D-(-)-2-Amino-5-phosphonopentanoic acid] (Abcam Biochemicals) were prepared fresh in artificial cerebral spinal fluid (ACSF) and added to solutions as indicated. D-AP5, the active isomer of AP5, was used to avoid potential variability associated with a racemic mixture. The behavioral experiments utilized mice that were intraperitoneal (i.p.) injected drug to more closely mimic the route of administration in humans. For the field potentials (FPs) recordings, after 20 min of stable baseline, drugs (ketamine, D-AP5) were applied for 30 min at rest and then one control stimulus was applied, after which there was no stimulation during a 1 h washout. Stimulation was resumed for 30 min after washout.

### BEHAVIOR

#### Novelty suppressed feeding

Mice were food deprived 24 h prior to testing. On testing day, mice were habituated to single housing in fresh home cages for 1 h. Mice were given an i.p. injection of vehicle (0.9% saline; *n* = 10) or ketamine (3 mg/kg; *n* = 10) and tested 30 min later. Small pieces of regular chow were placed in the center of a brightly lit open arena (42 cm × 42 cm). Mice were introduced into a corner of the arena and allowed to explore for up to 3 min. Latency to bite the food was recorded in seconds (s). To control for appetite level, mice were immediately removed from the test arena after consuming food or at the end of the 3 min trial and placed individually in the home cage where they were presented with a pre-weighed amount of chow for 5 min. The food was weighed at the end of the session and the amount consumed (difference) was recorded in grams (*g*).

#### Forced swim test

The same groups of mice were tested in the forced swim apparatus 24 h after vehicle or ketamine administration. Mice were habituated to the testing room for 1 h. Four liter beakers were filled with 3 L of water (24°C; changed between subjects) and a video camera was placed on the side of the beakers to record the session. Mice were placed in the water for 6 min. An observer blind to treatment scored seconds (s) spent immobile during the last 4 min of the test.

### EXTRACELLULAR FIELD POTENTIAL RECORDINGS

Hippocampal slices (400 μm) were prepared from 14 to 21 day (young) or 6–8 week (adult) old Sprague Dawley rats, as indicated. Animals were anesthetized with isoflurane and decapitated soon after the disappearance of corneal reflexes. The brain was removed, dissected, and then sliced using a vibratome (VT 1000S, Leica) in ice–cold dissection buffer containing the following (in mM): 2.6 KCl, 1.25 NaH_2_PO_4_, 26 NaHCO_3_, 0.5 CaCl_2_, 5 MgCl_2_, 212 sucrose, and 10 dextrose. Area CA3 was surgically removed from each slice immediately after sectioning. The slices were transferred into a reservoir chamber filled with ACSF containing the following (in mM): 124 NaCl, 5 KCl, 1.25 NaH_2_PO_4_, 26 NaHCO_3_, 2 CaCl_2_, 2 MgCl_2_, and 10 dextrose. Slices were allowed to recover for 2–3 h at 30°C. ACSF and dissection buffer were equilibrated with 95% O_2_ and 5% CO_2_. For recording, slices were transferred to a submerged recording chamber, maintained at 30°C, and perfused continuously with ACSF at a rate of 2–3 ml/min.

Field potentials were recorded with extracellular recording electrodes (1 MΩ) filled with ACSF and placed in stratum radiatum of area CA1. FPs were evoked by monophasic stimulation (duration, 200 μs) of Schaffer collateral/commissural afferents with a concentric bipolar tungsten stimulating electrode (Frederick Haer). Stable baseline responses were collected every 30 s using a stimulation intensity (10–30 μA), yielding 50–60% of the maximal response. FPs were filtered at 2 kHz and digitized at 10 kHz on a personal computer using custom software (LabVIEW, National Instruments). Synaptic strength was measured as the initial slope (10–40% of the rising phase) of the FP. The group data were analyzed as follows: (1) the initial slopes of the FP were expressed as percentages of the preconditioning baseline average; and (2) the time-matched, normalized data were averaged across experiments and expressed as mean ± SEM. Significant differences were determined by paired *t*-test, *p* < 0.05 was considered to represent significant differences.

### STATISTICS

For all experiments the data are represented as mean ± SEM. For the behavioral experiments, two-tailed *t*-tests were used to determine significance (*p* < 0.05). For the electrophysiology experiments, significant differences were determined by paired *t*-test, *p* < 0.05 was considered to represent significant differences.

## RESULTS

### KETAMINE DOES NOT TRIGGER A RAPID ANTIDEPRESSANT RESPONSE IN JUVENILE ANIMALS

**FIGURE 1:**
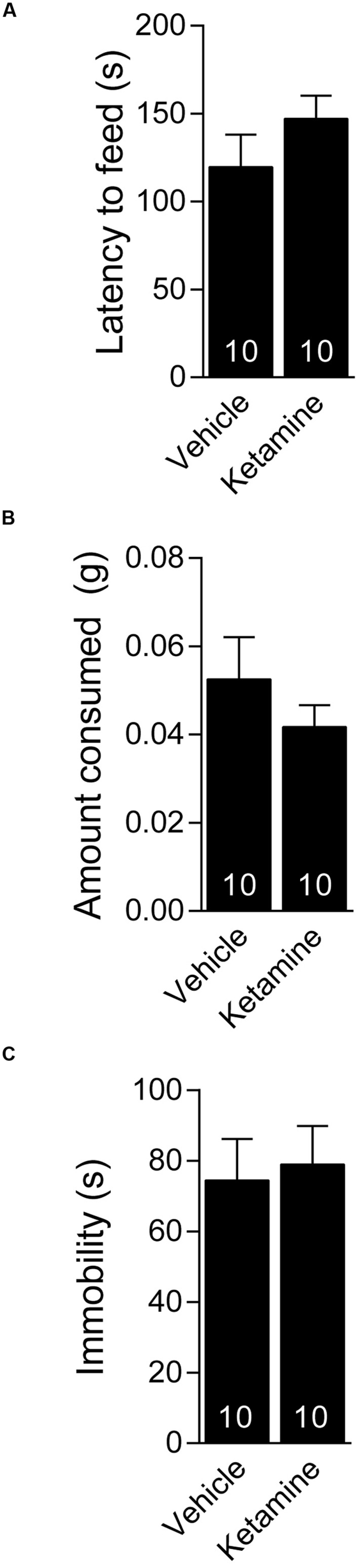
**Juvenile mice do not respond behaviorally to acute ketamine treatment. (A)** Four week old C57BL/6 male mice administered vehicle (saline) or ketamine (3 mg/kg) i.p. display similar latency to consume food in a novel environment. **(B)** Food consumption over a period of 5 min is indistinguishable between ketamine or vehicle-treated juveniles. **(C)** The same groups of mice tested 24 h after i.p. vehicle or ketamine treatment show comparable immobility in the forced swim test.

We investigated whether ketamine triggers an antidepressant response in 4 week old C57BL/6 juvenile mice. The juvenile mice were treated with 3.0 mg/kg ketamine (i.p.), a dose that triggers a rapid antidepressant response in young adult (6–8 week old) mice (Autry et al., 2011; Nosyreva et al., 2013) and examined 30 min later in the NSF test. We found no difference between the ketamine and vehicle treated mice in the latency to consume the food pellet in the juvenile mice suggesting that ketamine did not trigger an antidepressant response (**Figure 1A**). There was no difference in the amount of food consumed between the ketamine and vehicle treated groups, suggesting that the findings in the NSF test were not confounded by hunger (**Figure 1B**). To further examine the antidepressant effects of ketamine in juvenile mice, we tested the mice 24 h after ketamine treatment in the FST. We found that ketamine did not alter the immobility time in the FST further suggesting that it does not trigger a behavioral antidepressant response in juvenile mice (**Figure 1C**).

### KETAMINE APPLICATION AT REST DOES NOT ELICIT APPRECIABLE SYNAPTIC POTENTIATION IN SLICES FROM JUVENILE ANIMALS

**FIGURE 2:**
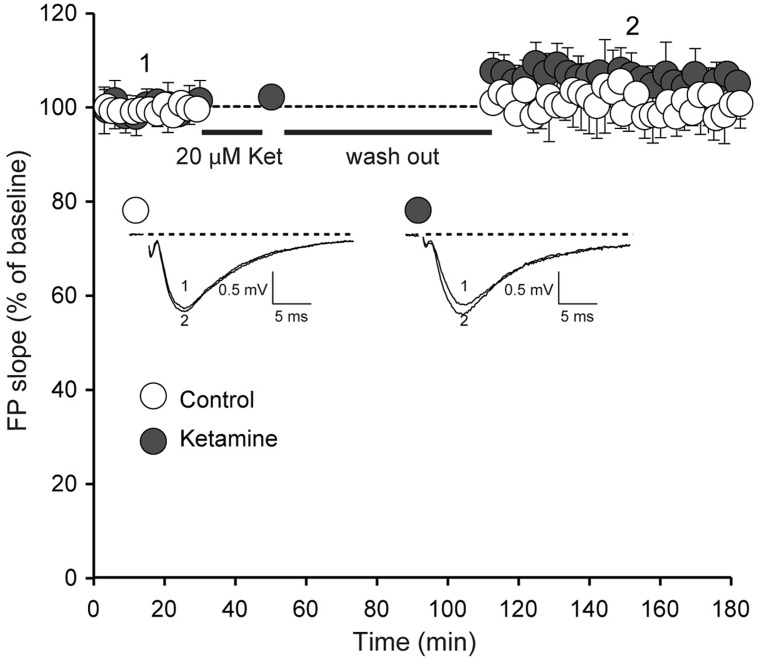
**Ketamine application does not induce significant synaptic strength potentiation in juvenile animals.** Field potentials (FPs) were recorded in control (*n* = 6) and ketamine-treated (20 μM) slices (*n* = 11), from 14-day-old rats. Initial FP slopes are plotted as a function of time (mean ± SEM). We did not observe significant changes of FP slopes compared to baseline after 90 min with or without application of ketamine (*p* = 0.097 and *p* = 0.24 respectively). Inset, Representative waveforms from control and ketamine-treated slices recorded at the times indicated by the numbers on the graph (1, 2).

**FIGURE 3:**
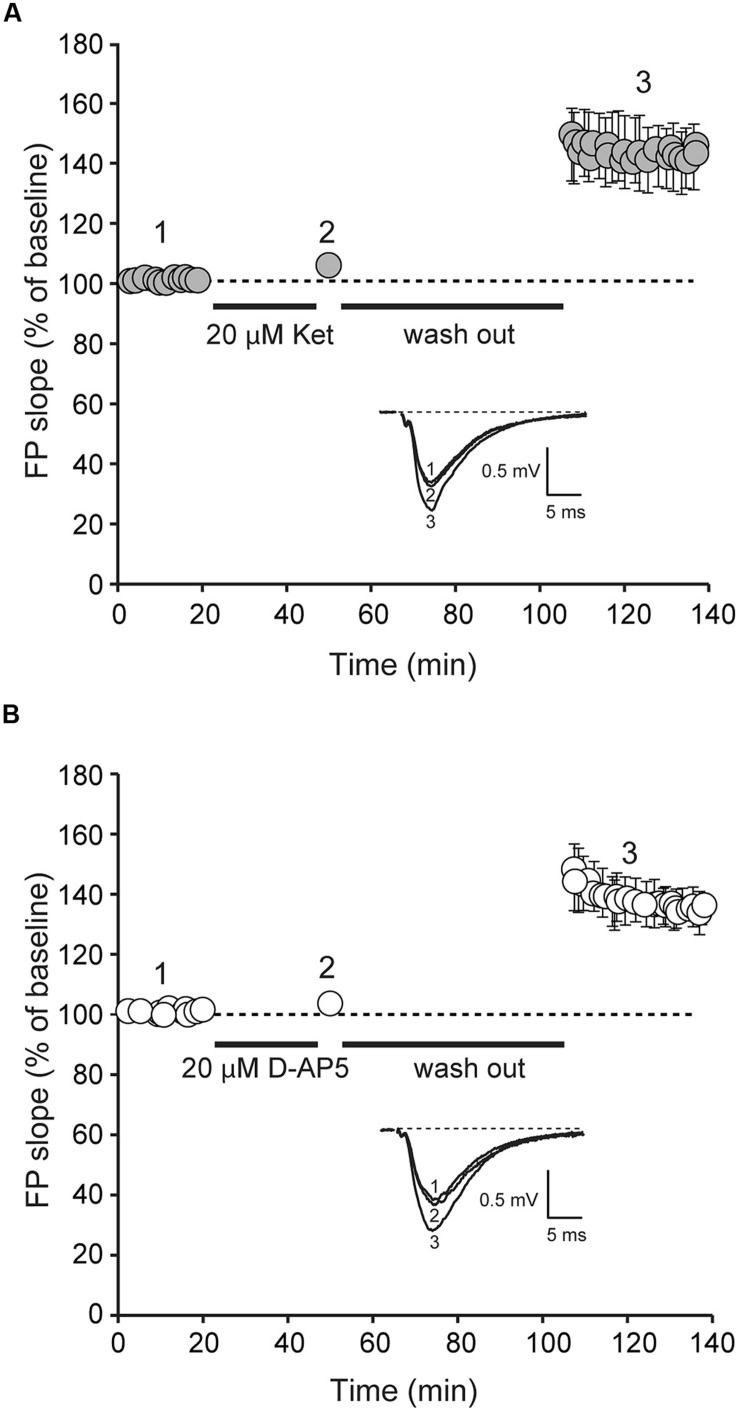
**Application of D-AP5 and ketamine at rest induces potentiation of AMPAR-mediated evoked neurotransmission.** Field potentials (FPs) were recorded from ketamine and D-AP5 treated slices from adult animals. Initial FP slopes are plotted as a function of time (mean ± SEM).** (A)** Application of ketamine (20 μM) induces significant increase in FP slopes (*n* = 8, *p* < 0.05).** (B)** Application of D-AP5 (20 μM) induces significant increase in FP slopes (*n* = 9, *p* < 0.05). Insets **(A,B)**, Representative waveforms from D-AP5 and ketamine-treated slices recorded at the times indicated by the numbers on the graph (1, 2).

We next examined whether ketamine elicits synaptic potentiation in hippocampal slices of juvenile mice. To study the effect of ketamine application on synaptic efficacy, we initially recorded baseline field excitatory postsynaptic potentials (fEPSPs) at a frequency of 0.03 Hz from the CA1 region of a hippocampal slice from 2 to 3 week old animals following extracellular stimulation of CA3 Schaffer collaterals. In the setting, following 30 min long recordings of the baseline synaptic responses, stimulation was stopped and 20 μM ketamine was applied for another 30 min. After subsequent wash out of ketamine we detected only a modest shift in baseline that did not reach significance (**Figure 2**). Previous work has shown that this stimulation paradigm elicits a significant potentiation in mature hippocampal slices (Autry et al., 2011; Nosyreva et al., 2013). To confirm these previous findings, we applied the same experimental protocol in developmentally mature hippocampal slices (6–8 weeks old) and found that ketamine triggered a robust potentiation that reached 44 ± 0.004% within 1 h after drug removal (**Figure 3A**). These data demonstrate that ketamine does not elicit synaptic potentiation in developmentally immature synaptic contact as it does in mature synapses demonstrating an age dependence for the plasticity mediated processes involved in ketamine action.

### APPLICATION OF THE COMPETITIVE NMDAR BLOCKER, D-AP5, AT REST ELICITS SYNAPTIC POTENTIATION IN MATURE SLICES

To determine whether the widely used competitive NMDAR antagonist, D-AP5, could elicit synaptic potentiation in developmentally mature hippocampal slices, we applied D-AP5 to hippocampal slices from 6 to 8 week old animals using the same protocol in **Figures 2** and **3A**. Under these conditions, we detected a robust potentiation (39 ± 0.006%) of synaptic efficacy following removal of D-AP5 (**Figure 3B**). This result indicates that the synaptic effect of ketamine at rest is not specific to the use-dependence of ketamine action and solely requires global suppression of NMDAR function at rest.

## DISCUSSION

In this study, we examined the antidepressant action of ketamine and its impact on synaptic transmission in juvenile animals and found an absence of the robust effect of ketamine on behavior and synaptic plasticity. This finding suggests that the antidepressant effects of ketamine strictly require formation of mature synaptic circuitry. As NMDAR activity can be detected soon after formation of initial synaptic contacts (Wu et al., 1996; Hanse et al., 2013), we hypothesize that the number and the strength of these synapses rather than the presence or absence of NMDARs are likely determinants of this form of synaptic plasticity. Previous studies have documented a robust increase in the number of synaptic contacts within the maturational time scale that we examined here. For instance, within the CA1 region of the hippocampus the rate of spontaneous miniature excitatory postsynaptic currents (mEPSC’s) increase fourfold over the first 2 months of development, indicating an increase in synaptic connectivity as well as a potential increase in the rate of spontaneous release per synapse (Hsia et al., 1998). As the ability of ketamine to elicit synaptic potentiation depends on its blockade of resting NMDAR mediated synaptic currents (Nosyreva et al., 2013; Gideons et al., 2014), the increase in mEPSC frequency during this developmental window, therefore, may render synaptic efficacy more susceptible to NMDA-mEPSC block. This premise may suggest the existence of a specific threshold for mEPSC activity to exert its effect on homeostatic synaptic plasticity. However, the developmental timeline for the emergence of the ketamine effect may also depend on a developmental switch in protein translation dependence of synaptic plasticity. Indeed, there is evidence that mGluR mediated long term synaptic depression (mGluR-LTD) in juvenile animals is independent of protein synthesis. However, in mature animals the same mGluR activation protocol elicits LTD in a manner that requires protein translation and a long-term decrease in AMPA receptor (AMPAR) surface expression (Nosyreva and Huber, 2005). In addition, it is possible that changes in NMDAR expression and their contribution to synaptic efficacy depending on developmental stages could impact the findings. However, at the hippocampal synapses in question, NMDAR mediated synaptic responses are detectable even at very early stages of synapse development (Liao et al., 1995; Choi et al., 2000) suggesting that lack of NMDARs is an unlikely scenario.

Our results also show that the competitive NMDAR antagonist, D-AP5, can elicit robust synaptic potentiation when applied to developmentally mature hippocampal slices. This finding correlates with the previously reported effect of another competitive NMDAR antagonist, CPP, to elicit a rapid antidepressant like response in mice (Autry et al., 2011). This finding that D-AP5 could elicit robust synaptic potentiation is important and in agreement with earlier work in which we could replicate the potentiation elicited by ketamine using another use dependent blocker, MK801. This result, while confirming the specificity of the effect to the NMDAR function, left open the question of whether this plasticity required the application of a use dependent blocker rather than global competitive blockade of NMDAR at the glutamate binding site. Use dependent blockers by their nature show higher efficacy to block NMDARs that are juxtaposed to presynaptic terminals with higher release probability (Hessler et al., 1993; Rosenmund et al., 1993). Therefore, it is possible that blockade of higher release probability synapses might be necessary for this effect as a gradual low frequency activation at the remaining synapses may be permissive. However, our results demonstrate that global NMDAR blockade can elicit a significant potentiation attesting that the functional subdivision of NMDAR activation patterns does not play a role in the regulation of this potentiation. It is important to indicate that earlier *in vitro* experiments have relied on AP-5 treatment to detect a postsynaptic protein translation and eEF2 kinase dependent scaling effect (Sutton et al., 2007). This earlier synaptic scaling effect bears the hallmarks of the potentiation we detect in acute hippocampal slices.

It would be interesting to know whether AP5 produces an antidepressant response. However, literature in the field has shown that peripheral administration of AP5 does not cross into the brain (Tonkiss and Rawlins, 1991; Coleman et al., 2013). Our choice of AP5 was based on its common use in examining NMDAR antagonism and thus addressing the question whether the potentiation we observed previously using non-competitive open channel blockers ketamine and MK-801 could be triggered by a competitive blocker. In addition, in lieu of AP5, we have previously shown that a competitive NMDAR antagonist CPP can exert a rapid antidepressant effect (Autry et al., 2011).

Antidepressants such as selective serotonin reuptake inhibitors (SSRIs) have no notable clinical effects in healthy subjects. However, in rodent models antidepressants including SSRIs and ketamine trigger ‘antidepressant-like’ effects in naïve animals (e.g., Lucki et al., 2001; Monteggia et al., 2004; Adachi et al., 2008; Maeng et al., 2008; Li et al., 2010; Autry et al., 2011; Nosyreva et al., 2013) as well as stressed animals in behavioral paradigms such as the FST and NSF tests. However, a recent study (Donahue et al., 2014) demonstrated that acute low dose of ketamine failed to attenuate chronic social defeat stress suggesting that ketamine action is not necessarily more valid in tests of chronic stress in elucidating the mechanism of antidepressant efficacy *per se*. Therefore, these data taken together with the fact that ketamine produces an antidepressant response in naïve adult animals led us to focus our study on naïve juvenile animals in the FST and NSF test.

Our findings demonstrating that ketamine does not trigger antidepressant responses or synaptic potentiation in juvenile animals suggest a developmental component to antidepressant efficacy. We also show that global blockade of NMDARs in developmentally mature hippocampal synapses are required for the antidepressant efficacy of ketamine. While clinical studies of ketamine’s action as an antidepressant have not been examined in juvenile patients with MDD, our findings suggest a potential limited therapeutic potential for younger patient populations.

## Conflict of Interest Statement

The authors declare that the research was conducted in the absence of any commercial or financial relationships that could be construed as a potential conflict of interest.
